# Spatial and temporal risk as drivers for adoption of foot and mouth disease vaccination

**DOI:** 10.1016/j.vaccine.2018.06.069

**Published:** 2018-08-09

**Authors:** Ashley F. Railey, Tiziana Lembo, Guy H. Palmer, Gabriel M. Shirima, Thomas L. Marsh

**Affiliations:** a Paul G. Allen School for Global Animal Health, Washington State University, USA; b Boyd Orr Centre for Population and Ecosystem Health; Institute of Biodiversity, Animal Health and Comparative Medicine; College of Medical, Veterinary and Life Sciences, University of Glasgow, Scotland, United Kingdom; c Nelson Mandela African Institution of Science and Technology, Tanzania; d School of Economic Sciences, Washington State University, USA

**Keywords:** Perceived risk, Spatial risk, Temporal risk, Uncertainty, Vaccination, Foot-and-mouth disease

## Abstract

Identifying the drivers of vaccine adoption decisions under varying levels of perceived disease risk and benefit provides insight into what can limit or enhance vaccination uptake. To address the relationship of perceived benefit relative to temporal and spatial risk, we surveyed 432 pastoralist households in northern Tanzania on vaccination for foot-and-mouth disease (FMD). Unlike human health vaccination decisions where beliefs regarding adverse, personal health effects factor heavily into perceived risk, decisions for animal vaccination focus disproportionately on dynamic risks to animal productivity. We extended a commonly used stated preference survey methodology, willingness to pay, to elicit responses for a routine vaccination strategy applied biannually and an emergency strategy applied in reaction to spatially variable, hypothetical outbreaks. Our results show that households place a higher value on vaccination as perceived risk and household capacity to cope with resource constraints increase, but that the episodic and unpredictable spatial and temporal spread of FMD contributes to increased levels of uncertainty regarding the benefit of vaccination. In addition, concerns regarding the performance of the vaccine underlie decisions for both routine and emergency vaccination, indicating a need for within community messaging and documentation of the household and population level benefits of FMD vaccination.

## Highlights

• The value of vaccination increases with perceived risk.
• Heightened proximity of risk introduces uncertainty.
• Household capacity to cope with resource constraints influences vaccination uptake.
• Concerns for vaccine efficacy undermine vaccination coverage.

## 1 Introduction

Uncertainty surrounding health decisions stems from unknown gains in personal wellbeing relative to the perceived costs of undertaking the intervention. This is specifically applicable to vaccination decisions. For example, the decision to be vaccinated against seasonal influenza weighs perception of individual risk of disease against the direct costs of the vaccination, the indirect costs of the time necessary to be vaccinated (lost opportunity), and any concerns about adverse vaccination effects [1]. The implications of individual vaccination in contributing to population immunity further complicates the decision. Importantly, perceptions of disease risk are dynamic and may markedly increase as disease outbreaks are reported closer to the individual [2], [3], [4]. However, by this time much of the potential for inducing population immunity is lost, and vaccination benefits may only extend to the recipient.

Understanding the drivers of vaccination decisions and how these are influenced by proximity of perceived risk is a significant gap in vaccine knowledge relevant to increasing vaccination and decreasing the burden of infectious disease. We chose to address this knowledge gap by estimating pastoralist adoption of a livestock vaccination against foot-and-mouth disease (FMD). Similar to seasonal influenza, FMD is episodic and not precisely predictable in either spatial or temporal spread or in its severity [5], thus creating uncertainty of disease risk. Furthermore, there are multiple FMD virus serotypes with each serotype characterized by evolving strains. FMD vaccines vary in their effectiveness depending on the “match” between the vaccine and the circulating serotype and strain [6], [7], require repeated immunization to achieve optimal protection, and are similar to seasonal influenza vaccines in having effects at both the individual and population levels [8], [9], [10]. Unlike human vaccination or vaccination for zoonotic livestock diseases that have human health implications, the decision to vaccinate for FMD solely fixates on livestock health, and thus focuses our analysis on externally influenced, dynamic risk perceptions [11]. Importantly, in households that are characterized by high dependence on livestock, vaccination decisions have broad impacts on household income and wealth, food security, and expenditures on human health and education [12]. For FMD specifically, reductions in milk production, lost animal draught power, and closure of livestock markets threaten household income and nutritional security [13].

We surveyed 432 pastoralist households in northern Tanzania to identify determinants of FMD vaccination decisions relative to temporal and spatial risk based on two immunization strategies. We extended a commonly accepted survey method for inferring preferences, willingness to pay (WTP), to elicit decision responses for two hypothetical vaccination scenarios. The first is “routine” vaccination in which households would vaccinate cattle biannually, a proactive and planned approach that would support immunity at population scale. The second is “emergency” vaccination in which households would vaccinate in the face of a current nearby outbreak, a situation that presents heightened, individualized risk introduced by spatial proximity and temporal immediacy. In each scenario, the stated efficacy of the vaccine was also varied to reflect the uncertainty of the vaccine matching process and to assess sensitivity to improvements in vaccine risk reduction. Herein we present the results of the study and discuss the findings in the context of identifying approaches to influence household vaccine uptake and subsequent improved disease control.

## 2 Materials and methods

### 2.1 Household survey and data

**Fig. 1:**
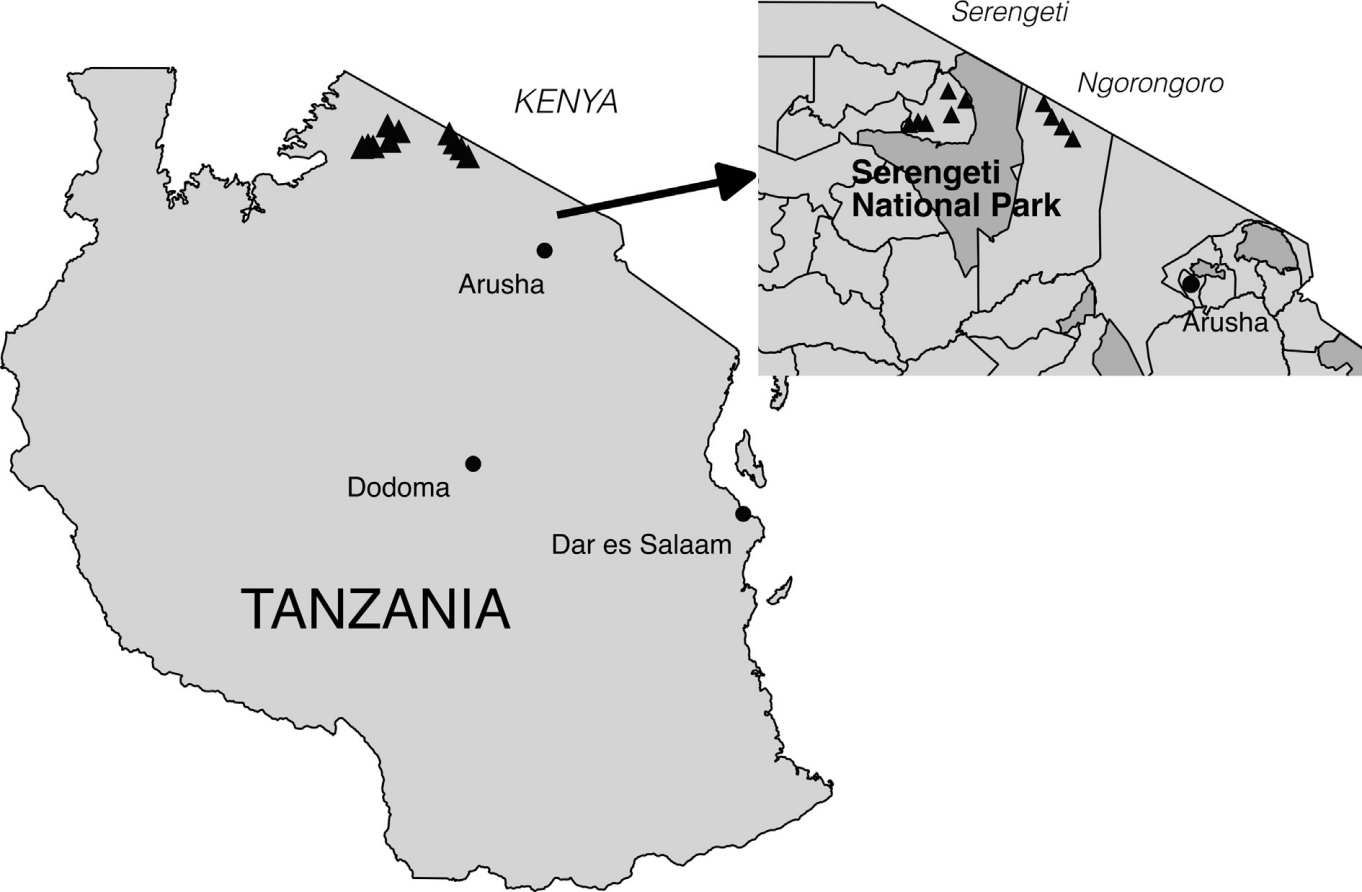
Location of the 10 study sites (triangles) within the Serengeti and Ngorongoro districts in relation to major Tanzanian cities (circles) and parks (dark grey).

The survey questionnaire that was used for data collection targeted key decision makers in cattle owning households to identify behavioral responses and to increase accuracy and precision of those responses. The cross-sectional survey was conducted in April through July 2016 in the Serengeti and Ngorongoro districts of northern Tanzania (Fig. 1) and contained questions designed to capture household characteristics hypothesized to influence vaccination WTP, including household demographics, livestock management practices, and knowledge of and history with FMD. Within the two districts, a two-stage sampling procedure randomly selected first clusters, then households with the Serengeti district more intensively sampled for analysis purposes (Supplementary material) [14]. Design and piloting of the survey instrument followed standard statistical practices [14]. Informed consent was obtained after the nature and possible consequences of the study had been explained by local enumerators who were trained and monitored throughout the collection process.

Households in both study districts engage in livestock and agricultural activities for subsistence and income, with some additionally earning income from off-farm activities (Table 1). Households practice open grazing and own 20 cows compared to the national average of 4 cows [15], in addition to owning sheep, goats, and poultry. Consistent with previous estimates of FMD occurrence in these areas, 69% of the households reported infection within the past year [16] and expected reductions in milk production during outbreaks. All households recognized the clinical signs of FMD, but of the 19% who had vaccinated for any livestock disease in the past year, none reported vaccinating for FMD. This reflects the situation of FMD in East Africa as characterized by poor surveillance systems and limited availability of FMD vaccines (Supplementary material).

**Table 1:** Descriptive statistics for variables used in analysis.

Variable	Mean‡ or proportion	Std. Dev.	Median	Min	Max
No formal education	0.16	0.36	0	0	1.0
Income					
Monthly off-farm (≤25,000 Tsh)	0.74	0.46	1.0	0	1.0
Monthly off-farm (25–100,000)	0.13	0.34	0	0	1.0
Monthly off-farm (>100,000)	0.13	0.33	0	0	1.0
Seasonal crops (≤100,000 Tsh)	0.67	0.47	1.0	0	1.0
Seasonal crops (100–500,000)	0.16	0.36	0	0	1.0
Seasonal crops (>500,000)	0.18	0.38	0	0	1.0
Herd size‡	42	59	20.0	1.0	530
Expected milk loss (in liters per cow)‡	0.70	0.59	0.61	0	5.0
Cattle sold in the past year‡	5.9	6.7	2.0	0	21
FMD experienced in past year	0.69	0.46	1.0	0	1.0
Vaccinated for any cattle disease in past year	0.19	0.40	0	0	1.0
Use government vet	0.34	0.48	0	0	1.0
Vaccine efficacy of 50%	0.45	0.49	0	0	1.0
Male head of household	0.84	0.36	1.0	0	1.0
50% efficacy*male head of household	0.60	0.49	0	0	1.0
Serengeti district	0.59	0.49	1.0	0	1.0
Outbreak @ Neighbor	0.57	0.50	1.0	0	1.0
n = 432					

‡ mean of continuous variables.

### 2.2 Double bounded dichotomous choice contingent valuation

The absence of FMD vaccines in Tanzania during the time of the study led to the use of the stated preference methodology to infer the value households place on vaccination. We used the double bounded dichotomous choice contingent valuation method [17], [18], which is a standard survey approach that can jointly analyze willingness to pay to adopt a product and determine factors underlying adoption behavior. For both the routine and emergency vaccination strategies, households received an initial, binary choice (‘yes/no’) question eliciting WTP for a single vaccine dose if it protects one cow from FMD over a 6-month period. Households then received a follow up, second binary choice question raising or lowering the offered price depending on the response to the first. Each respondent received the same initial bid price, 2000 Tsh (USD 0.95) for the routine vaccine and 4000 Tsh (USD 1.90) for the emergency vaccine, with the follow up bids for the routine vaccines ranging between 500 Tsh (USD 0.24) and 3500 Tsh (USD 1.67), and the emergency between 500 (USD 0.24) and 7500 Tsh (USD 3.57).

The binary choice question format of this model is cognitively easier than other question designs by removing the burden on the respondent to formulate a price or choose between multiple hypothetical scenarios [19]. Bias can exist with the double bounded model if the household’s WTP value changes between responding to the first bid and the second, follow up bid question [20]. Use of the interval data model can remove some of this concern by providing robust WTP estimates [21]. Additional pretesting of bid levels and referencing related vaccine prices reduces large deviations from the potential market price range [22]. Compared to the single bounded model that only asks one binary choice question, the gain in asymptotic efficiency of the double bounded approach outweighs the potential bias from anchoring on the initial bid [23].

### 2.3 Empirical model

The empirical strategy is modeled according to expected utility theory and estimated using the maximum likelihood estimator (Supplementary material). The average emergency WTP is anticipated to be higher than the routine WTP by an amount proportional to the change in risk reduction between the two [17], [18]. In an emergency situation, the immediacy of the risk increases the individual perceived value of vaccination, whereas the delayed reward of routine vaccination reduces its relative value [24]. However, households can reasonably plan for biannual vaccination costs whereas the unanticipated income shock in an emergency, coupled with increased susceptibility, introduces uncertainty towards the relative gain from vaccination [25]. We presented the routine question first to provide a baseline perception of risk, followed by the question regarding emergency vaccination.

To reflect the complexity of the vaccine matching process and assess sensitivity to improvements in vaccine-induced risk reduction, households were randomly assigned a stated vaccine efficacy of 50% or 100%. The emergency vaccination question was then conditioned on outbreak distance, either with a neighbor (1 km radius) or at the village level (5 km radius). The proximity of neighbors within one kilometer for all households in the study is assumed to imply immediate, unavoidable exposure. An outbreak at the village level attempts to alleviate perceptions of disease risk through the possibility that exposure has not occurred. The 5 km radius roughly follows village boundaries and was provided to the respondent when the household had difficulty conceptualizing village proximity.

## 3 Results

### 3.1 Routine versus emergency vaccination

**Fig. 2:**
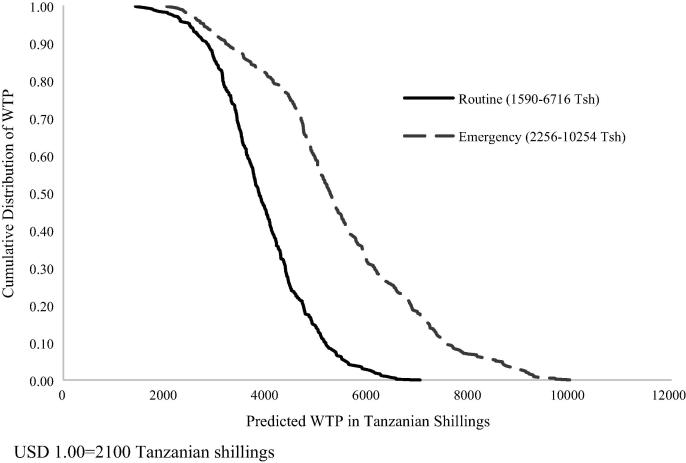
Distribution of Willingness to Pay Values.

Following theoretical and practical guidance on perceived risk under routine and emergency vaccination scenarios, we expected differential behavioral processes between the two. To assess the appropriateness of modeling the two strategies separately, we performed a likelihood ratio test. The test rejected the estimation of both vaccination strategies jointly in favor of separate models (Supplementary material), supporting the concept that different decision-making processes influence each type of vaccination. Estimation of the two models separately then revealed a higher mean WTP and more variation in the distribution for emergency vaccination relative to routine, further providing fundamental empirical evidence that household behavior is differentiated between routine and emergency strategies (Fig. 2). The mean WTP of emergency vaccination was around 5400 Tsh (95% CI: 3551, 7271 Tsh; USD 1.69, 3.46) and about 3900 Tsh for a routine strategy (95% CI: 2555, 5217 Tsh; USD 1.22, 2.48).

### 3.2 Perceived risk sensitivity

Separate WTP distributions for the two strategies in and of themselves are not sufficient to conclude that households accurately valued the varying levels of uncertainty and risk associated with FMD vaccination. To support this conclusion, we would expect that responses be consistent and sensitive to marginal changes in risk. We therefore first examined the effect of an increase in vaccine bid price on the probability of adopting vaccination. We found that as the bid price increased for both strategies, the probability of vaccine acceptance decreased (Table 2), consistent with theoretical expectations [19]. We next compared the average WTP values for each strategy with the calculated change in perceived risk reduction between routine and emergency vaccination (Supplementary material). Also following theoretical expectations [17], [18], we found the marginal change in risk reduction going from a routine to an emergency strategy to be of comparable magnitude to the change in WTP values, with the risk reduction from an emergency strategy to be higher than a routine strategy.

**Table 2:** Probability of responding 'yes' to each bid (in Tsh).

Routine vaccination bid	Probability (%)	Emergency vaccination bid	Probability (%)
500	94	500	89
1000	82	2000	81
1500	58	3500	69
2000	48	4000	65
2500	38	4500	60
3000	29	5000	55
3500	5	7500	30

USD 1.00 = 2100 Tanzanian shillings.

For perceptions of risk from increased susceptibility, we found an outbreak with a neighbor compared to an outbreak at the village level presented no difference in the value of vaccination. Altering the vaccine efficacy level by itself did not influence vaccine valuation for either strategy. Contrary to expectations, households that received a vaccine of 50% stated efficacy did not value vaccination differently than those provided with a vaccine of 100% stated efficacy. However, interacting efficacy level (50 or 100%) with the head of household gender revealed that a male head of household that received the vaccine of 50% efficacy would pay less than a female head of households for either efficacy level and less than a male head of household receiving a vaccine of 100% efficacy (routine -1458 Tsh; USD 0.69; emergency −2718 Tsh; USD 1.29).

### 3.3 Vaccination determinants

As expected, the drivers behind routine and emergency vaccination adoption also differed (Table 3). For any health expenditure decision, diversity and liquidity of household income portfolios affects household capacity to invest in proactive measures, exemplified by routine vaccination, and adjust to near term threats and shocks, represented by emergency vaccination and the risk of major disease outbreaks and loss [26]. Our results show income apart from livestock is a primary determinant of emergency vaccination, while additionally influencing routine adoption but at a lower magnitude. Compared to households with no off-farm income, households with higher levels of off-farm income reported higher valuation of routine (1022 Tsh; USD 0.49) and emergency vaccinations (1962 and 1763 Tsh; USD 0/93/0.84). Diversity of income had varying effects on willingness to pay for both strategies. Compared to households with no crop income the prior season, earning some seasonal crop income (100 to 500,000 Tsh; USD 47.61–238.10) increased both routine (1635 Tsh; USD 0.78) and emergency WTP (2294 Tsh; USD 1.09). Being in the highest income bracket from crop sales in the prior season (>500,000 Tsh; USD 238.10) had no effect on adoption for either vaccine strategy.

**Table 3:** Vaccination determinants.

Variable	Routine marginal effects (CI 95%)	P value	Emergency marginal effects (CI 95%)	P value
Education (0 = Formal; 1 = No Formal)	681 (−7, 1356)	0.096	655 (−369, 1679)	0.295
Income				
Off-Farm (≤25,000 Tsh)	Base case			
Off-Farm (25–100,000)	589 (−34, 1213)	0.119	1962 (835, 3090)	0.004
Off-Farm (>100,000)	1022 (360, 1685)	0.010	1763 (672, 2854)	0.007
Crops (≤100,000 Tsh)	Base case			
Crops (100–500,000)	1635 (806, 2465)	0.001	2294 (1034, 3554)	0.003
Crops (>500,000)	−445 (−1067, 176)	0.237	−403 (−1513, 3554)	0.552
Herd Size^a^	26 (−192, 243)	0.846	42 (−348, 432)	0.859
Expected Milk Loss (in liters per cow)	306 (−94, 707)	0.207	423 (−205, 1051)	0.270
Cattle sold in past year	36 (−0.33, 71)	0.096	11 (−48, 71)	0.753
FMD experience in past year (0 = No; 1 = Yes)	−241 (751, 270)	0.439	−283 (−1156, 590)	0.595
Vaccinated for any cattle disease in past year (0 = No; 1 = Yes)	−247 (−795, 299)	0.457	216 (−754, 1186)	0.715
Use of government vet (0 = No; 1 = Yes)	−663 (−1113, −214)	0.014	−1817 (−2626, −1008)	0.001
Vaccine efficacy (0 = 100%; 1 = 50%)	1573 (370, 2778)	0.031	2318 (107, 4529)	0.085
Gender (0 = Female; 1 = Male)	1031 (321, 1740)	0.016	857 (−478, 2192)	0.292
Gender*efficacy (0 = 100%; 1 = 50%)	−1458 (−2740, −174)	0.060	−2737 (−5066, 406)	0.053
District (0 = Ngorongoro; 1 = Serengeti)	−270 (−751, 212)	0.358	94 (−779, 967)	0.860
Outbreak (0 = Village; 1=@Neighbor)			−476 (−1244, 293)	0.314
Log Likelihood	−415		−498	
Chi-2 Statistic	39.09		41.87	

USD 1.00 = 2100 Tanzanian shillings.

a Log of variable.

Beyond income, we assessed the role of exogenously determined motivations to vaccinate by including variables on whether a household receives livestock health information from a government veterinarian and the level of formal education of the head of household [27]. We found that households citing the government veterinarian as their main source of information (35% of households) reported lower WTP values than those relying on other sources (−663 Tsh; USD 0.32), with augmented effects for the emergency vaccine (−1817 Tsh; USD 0.86). Similarly, results on the education variable offered corresponding evidence that households maintain negative perceptions towards vaccination. We would expect formal education to be associated with adoption of disease prevention practices. Instead we found no effect of education on emergency vaccination, and, for a routine vaccination, head of households with no formal education (15% of households) reported higher WTP values than those with formal education (681 Tsh; USD 0.32). Use of government veterinarians and educational attainment were uncorrelated across income levels and villages.

## 4 Discussion

In agreement with the human health literature, individual risk perceptions affect the valuation of vaccination [28], [29]. Households place a higher value on vaccines when the threat of disease is perceived as immediate. However, concerns about vaccine efficacy and the vaccination process also factor into decisions to vaccinate with an augmented effect in an emergency situation. Unlike human vaccination or vaccination for zoonotic diseases, FMD vaccination minimizes perception of adverse side effects for the vaccinated individual but retains the impact on household welfare in terms of expenditures, loss of income risk, and coping with resource constraints. This is supported by three main findings in our study: (i) income is a major determinant in valuing both routine and emergency vaccination decisions; (ii) diversity of income promotes vaccination uptake (at least to a point); and (iii) there is a constant increased willingness to pay for emergency over routine vaccination. The fact that complete dependence on livestock income does not consistently promote vaccination further suggests income liquidity and diversity are important to facilitate risk responsiveness [30].

Compared to routine vaccination, weighing an unexpected shock with perceptions of susceptibility and disease severity increases decision uncertainty for emergency vaccination, as reflected by the greater variation in WTP. Similar to human seasonal influenza vaccination, imprecise spatial and temporal risk and disease severity are compounded in an emergency situation and confounds perceptions on the overall gain from vaccination relative to disease impact. As with most vaccines, FMD vaccination requires 7 to 14 days to provide protective immunity [31]. Households do not need to know the precise temporal or spatial information required to induce protection to understand that dense contact networks [32] and the difficulty of self-imposing movement restrictions [33] in pastoralist communities makes the likelihood of near immediate exposure almost certain if an outbreak is nearby. To support this point, we found no variation in the value of vaccination between offering an emergency vaccine when an outbreak is with a neighbor (1 km radius) or at the village level (5 km radius). Subsequently, for an emergency vaccine, the perceived risk is consistently higher, and the wider variation in WTP values reflects greater uncertainty about perceived gains from vaccination.

In both vaccination scenarios, uncertainty about the effectiveness of vaccination in preventing disease underlies the decision process. This includes doubts about vaccine effectiveness that are common to both human and animal vaccines. Governmental and non-governmental professional institutions attempt to alleviate concerns about effectiveness relative to cost and risk, but similar to influenza, these professional sources often struggle to fully overcome individual or community uncertainty. Pastoralist’s inexperience with FMD vaccines generally and with appropriately strain matched vaccines specifically could contribute to uncertainty in the value of vaccination, even though FMD is a common, episodic disease familiar to the community. This resembles concerns for seasonal influenza about the match of the vaccine strain to the circulating strain [34], [35]. Gender-based differences in WTP may reveal this, whereas men have more experience than women with non-poultry livestock vaccination and appropriately demonstrate sensitivity to improvements in vaccine quality. Furthermore, the disconnection between educational attainment and a positive decision for vaccination, may explain this, as informed households would rationally be less likely to adopt what are perceived as ineffective vaccines. These differences may also reflect overall lack of experience with vaccines. In the few households that had vaccinated for any livestock disease in the past year there was no influence on WTP for either FMD vaccination strategy. Lack of positive experience coupled with a poor understanding about the value of population level immunity—a concern shared with adoption of human vaccines, including seasonal influenza—indicates the relative benefits of vaccination remain imprecise.

For any vaccination strategy, we emphasize the need for clear messages about the public and private benefits of vaccination. Households seem to apprehend the presence or absence of direct FMD risk and accurately valued vaccination relative to risk. However, similar to other animal vaccination WTP studies, this relationship between perceived disease risk and WTP for effective vaccines [36] coupled with inadequate understanding of vaccines and population level effects [37], [38] suggests households need additional information and assurance that the benefits of the chosen vaccination strategy exceed the costs. Clearly communicating the need to vaccinate early, prior to local outbreaks, along with presenting an effective and serotype specific vaccine priced to capture the most economically vulnerable populations will help decrease the perceived risk of vaccination. In light of the limited positive influence on adoption from professionals, consistent with prior human and animal vaccination studies [12], [39], [40], [41], dissemination of vaccine knowledge through informal social networks is critical in overcoming existing negative expectations. Improved surveillance to detect circulating FMD serotypes and strains and vaccines that are better matched to local strains should reduce household vaccination uncertainty and increase vaccine uptake.

Our research is limited to eliciting stated preferences for vaccines that may differ from actual market outcomes and activities. Additional investigation into vaccine attributes may provide more precise estimates on specific vaccine qualities and delivery options that will further enhance uptake. Specifying outbreak distance with respect to context-specific herd contact distances may also increase our knowledge on thresholds to risk perceptions. Finally, access to panel data or responses directly before, during, and after FMD outbreaks would improve documentation of how perceptions of risk change with respect to real-time temporal immediacy.
