# Supplementary material for: Spatial and temporal risk as drivers for adoption of foot and mouth disease vaccination
Source: Vaccine. 2018 Aug 9;36(33):5077–83. doi: 10.1016/j.vaccine.2018.06.069 (PMC6073883; doi:10.1016/j.vaccine.2018.06.069)
Supplement: Supplementary Data 1 [file mmc1.docx]

Spatial and temporal risk as drivers for adoption of foot and mouth disease vaccination

**Supplementary Material**

*Study Design*

We used a multi-stage random sampling design. Households were within the Serengeti district and the Loliondo Game Control Area of the Ngorongoro district in the Mara and Arusha regions respectively. We chose households within the two regions by first defining logistically feasible wards, from which we randomly drew sub-villages, then randomly selected cattle owning households for a total sample size of 500 households. We do not distinguish between types of cattle owning households, but refer to all households as ‘pastoralist,’ defined as those involved in livestock raising practices. Based on the estimates of cattle owning populations in the two survey districts, we chose six villages in the Serengeti and four in the Ngorongoro (~300 and 200 households respectively). To obtain a sampling frame for each sub-village of households, village leaders provided a list of cattle owning households and then participated in randomly selecting households. Enumerators fluent in the local language (Swahili, in addition to either Kuria or Maa) were accompanied by community leaders and supervised throughout the survey. Each survey averaged 45-60 minutes. The head of household, or respondents 18 or older in absence of the head, responded to the questions. The survey included a confidentiality agreement and households could opt out of the survey at any point. Households were compensated for their time with an in-kind gift. Additional description of variables used in the analysis and summary statistics are in Tables S1 and S2.

Of the 500 households, 432 households responded to the vaccine willingness to pay questions for a non-response rate of 14 percent. We addressed nonresponses by following up with households three times and using cellphones to call absent heads of household.

*Foot-and-mouth disease in Tanzania*

Foot-and-mouth disease (FMD) is a highly contagious viral disease that affects cloven hoofed animals, including cattle, sheep, and goats. The disease is characterized by fever, lameness, and vesicular lesions on the feet, mouth, tongue, snout, and mammary glands of infected animals. Few mortalities result from FMD, but morbidity rates are high. Worldwide there are seven FMD serotypes, of which four are found to be circulating in Tanzania (O, A, SAT1, and SAT2) [1]. Each serotype additionally has variable intra-typic variants that limits the ability of vaccination with one serotype of FMDV to protect against other serotypes or even sub-types within the same serotype.

Control of FMD can be accomplished using both prophylactic (“routine”) and /or emergency vaccination strategies. Prophylactic vaccines maintain a minimum potency of 3 PD_50_ and require a booster at around 4-6 weeks, followed by subsequent vaccinations every 4-6 months depending upon the species and epidemiological conditions [2]. Emergency vaccines additionally can be of minimum potency (3 PD_50)_, but preferably should be of higher potency (>6 PD_50_) for wider immunity and rapid onset [3]. For either vaccination strategy, monovalent or polyvalent vaccines exist. In Africa, bivalent vaccines (SAT1 and SAT2) and trivalent vaccines with the SATs, O, and A are used [3]. However, the use of low-potency vaccines and the lack of surveillance systems to map serotype outbreaks has resulted in insufficient vaccine protection against clinical signs in Tanzania and Africa more broadly. In Tanzania, FMD vaccines have traditionally been produced outside of the country, and, thus, often fail to match the circulating serotypes.

*Double-Bounded Dichotomous-Choice Contingent Valuation Method*

To assess household stated preferences for vaccination strategies we used a double-bounded dichotomous-choice contingent valuation method with a maximum likelihood estimation. This method is widely used for private and public non-market goods. The survey provided respondents with separate questions for the routine and for the emergency vaccination scenarios and specified valuation for one cow, as opposed to vaccinating the entire herd. For both the routine and emergency vaccination strategies, households received an initial, binary choice (‘yes/no’) question asking if a household would be willing to pay (WTP) for a single vaccine dose if it protects one cow from FMD over a 6 month period. The emergency vaccine question further stated that an outbreak had occurred either with a neighbor or at the village (5km) level. Households then received a follow up question posing the same question but raising or lowering the bid to be paid based on the response to the first bid question.

*Empirical Issues*

Potential limitations of the double-bounded model involve concerns for response consistency between the first and second follow up bids for each vaccination scenario. Sensitivity to changes in disease risk between the routine and emergency vaccination questions and vaccine efficacy levels is necessary for theoretically consistent responses [4,5]. Sensitivity to risk refers to the ability of the household to react to the health risk reduction in a manner that is proportional to the proposed reduction in risk [5].

For consistency between the first and second follow-up bid questions, we assumed the respondent answers both questions with the same or similar WTP value in mind. The gain in efficiency of the double-bounded structure and robustness with interval data estimation outweigh the potential bias [6,7]. To further abate anchoring on the first bid for both scenarios, bid values were pretested for question clarity and construct validity. We consulted local prices of related livestock treatments and vaccines, as well as FMD vaccine prices in other countries, to establish bids consistent with local market price ranges [8]. The actual market price for a FMD vaccine in other countries ranges from USD 0.30 to USD 9.00 [9]. To assess protest bids, or scenario rejection, households that responded ‘no’ to the initial and follow-up bid received another question regarding reasons for non-payment as a debriefing measure and check for understanding. Responses stating the ‘government should pay’ or ‘I do not vaccinate’ were considered protest bidders or scenario rejection. Only two households fit this definition.

Next, we tested using models with non-pooled data relative to a model with pooled data from the routine and emergency strategy scenarios by performing a likelihood ratio test (LR). The unrestricted, non-pooled estimate is the composite log-likelihood of the two models estimated separately and the restricted is the log-likelihood of pooling the responses into a single model. We took two times the difference in the log-likelihoods of the unrestricted (${\hat{\theta}_{n}}^{U}$) and restricted (${\hat{\theta}_{n}}^{R}$) [10]:

$\lambda=2(L({\hat{\theta}_{n}}^{U})-L({\hat{\theta}_{n}}^{R})$)

We rejected the null hypothesis that the parameter estimates for the two models are the same. The log-likelihood of the restricted model was -932.17. The log-likelihood of the routine vaccine model was -415.97 and the emergency was -498.34 for an unrestricted log-likelihood of -914.31. The resulting test statistic 35.72 exceeded that of the Chi2 critical value at 14 degrees of freedom and 0.05 probability.

We then evaluated sensitivity of risk reduction between a routine and emergency vaccination scenario by comparing the WTP ratio with the relative risk ratio. The risk of infection should increase in an emergency situation relative to a routine situation causing WTP to increase in the amount of the change in risk reduction [4,5], such that:

$${WTP}_{E}>{WTP}_{R}$$

The consistency of the marginal value of the relative risk reduction in health, or the change from a routine to emergency strategy, is assessed with respect to the value of statistical life (VSL) of one cow. In our analysis, the value of statistical life of a cow can be represented by the market value of a cow. The perceived risk reduction *r_i_* for each strategy is then calculated by taking the average WTP for either vaccination divided by the average *VSL* of a cow:

$$r_{R}=\frac{{WTP}_{R}}{VSL}$$

$$r_{E}=\frac{{WTP}_{E}}{VSL}$$

In theory, the willingness to pay for a small disease risk reduction in the health of one cow should be strictly positive and proportional to the magnitude of the risk reduction. Or rather, the higher WTP for the emergency vaccine relative to the routine vaccine should yield a slightly larger perceived risk reduction.

$${WTP}_{E}>{WTP}_{R}\Longleftrightarrow r_{E}>r_{R}$$

The marginal value in perceived change in risk from a routine to an emergency vaccination strategy should then be positive:

$$\frac{r_{E}-r_{R}}{r_{R}}>0$$

We used the market value of a head of cattle in northern Tanzania, ranging from 200-400,000 Tsh (USD 95.00-190.00) and an average vaccination cost at both 2000 Tsh (USD 0.95) and 4000 Tsh (USD 1.90), to assess the expected bounds on a relative risk reduction. The expected risk reduction ranges from 0.005 to 0.02 assuming a constant *VSL*. We could estimate our empirical perceived risk reductions using the household reported average cattle market price of 330,000 Tsh (USD 157.14), ${WTP}_{E}$of 5400 Tsh, and ${WTP}_{R}$ of 3900 Tsh. The risk reduction for an emergency vaccination strategy (0.016) was higher than the routine (0.012). The marginal value of perceived change in risk of 0.33 corresponded in magnitude to the proportional increase in WTP values of 0.38. The consistency between perceived risk and vaccination value align with ratios calculated from market values

Finally, the conditioning of the vaccination strategies on 50 and 100 percent efficacy is both an important policy distinction and measure of sensitivity to vaccine quality [4]. Households were randomly assigned a stated vaccine efficacy of 50 percent or 100 percent, implying a risk reduction by a factor of 2 from 1/100 (50 percent) to 1/50 (100 percent) for one dose of FMD vaccine with a 50-head herd of cattle. We tested a range of efficacy levels (70 vs. 100 percent; 60 vs. 90 percent) before selecting 50 and 100 percent to provide sufficient difference in scope.

*Econometric Model*

We modeled separate functions for the routine vaccination strategy, *R*, and emergency vaccination strategy, *E*, for each household *i.* Following theoretical understandings of WTP for health risk reductions [4,5], we hypothesized the occurrence of an outbreak resulting in a higher WTP for emergency vaccines than routine vaccines. Vaccination is a function of the bid combination $B_{i}$ presented to the household, a vector of observable household characteristics including the vaccine efficacy presented to the household *z_i_* and the outbreak threshold presented in an emergency, and unobservable random characteristics $\epsilon$. Assuming linearity in the parameters, the relationship can be modeled as:

$$y_{iR}\left( B_{i}, Z_{i} \right)=\alpha+\rho B_{i}+{\lambda'z}_{i}+\epsilon_{i}$$

$$y_{iE}\left( B_{i}, Z_{i} \right)=\alpha+\rho B_{i}+\lambda'z_{i}+\epsilon_{i}$$

$$y_{iE}\left( B_{i}, Z_{i} \right)-y_{iR}\left( B_{i}, Z_{i} \right)>0$$

where $\alpha$, $\rho$ and $\lambda$ unknown parameters to be estimated and WTP for an emergency strategy is greater than WTP for a routine strategy. We assumed the random error $\epsilon$ follows a cumulative logistic distribution with a mean of zero and a variance of $\sigma^{2}$.

The empirical model imposes a double-bounded sequence of decisions on willingness to pay whereby the WTP $y_{i}$ is a latent variable not directly observed. We suppressed the household subscript *i* for simplicity. Letting B_I_ be the initial bid, followed by B_high_ for the second bid following a ‘yes’ response to the first bid and B_low_ be the bid offered after an initial ‘no’ response. A ‘no-no’ response indicated the household WTP lies below the offered bids (-∞, B_low_), while a ‘yes-yes’ response suggested the value is either the highest bid offered, or above that bid (B_high_, ∞). The ‘no-yes’ and ‘yes-no’ responses imply the household’s maximum WTP fell between either of these two bid amounts [B_low_, B_I_) and [B_I_, B_high_) respectively. A household’s true WTP value can be enclosed within specified intervals and resulting in four discrete outcomes of the bidding process:

$$Pr(y)=\left[ \begin{aligned} I_{nn} if WTP_{i}<B_{low} \\ I_{ny} if B_{low}\leq WTP_{i}<B_{I} \\ I_{yn} if B_{I}\leq WTP_{i}<B_{high} \\ I_{yy} if WTP_{i}\geq B_{high} \end{aligned} \right.$$

whereby $I_{nn}$, $I_{ny}$, $I_{yn}$, $I_{yy}$are binary-valued indicator variables denoted as one if the argument is true, and zero otherwise. The individual WTP outcomes are based on the random utility model such that a utility maximizing household *i* will accept the proposed bid for either vaccine if the household believes it will be better off by paying for the vaccine than foregoing the vaccine. The utility the household receives from any of the four outcomes can be modeled as a probability statement,

$${Pr(I}_{nn}=1)=F\left( \alpha+\rho B_{low}+\lambda'z_{i}+\epsilon_{i} \right)$$

$${Pr(I}_{ny}=1)=F\left( \alpha+\rho B_{i}+\lambda'z_{i}+\epsilon_{i} \right)-F\left( \alpha-B_{low}+\lambda'z_{i}+\epsilon_{i} \right)$$

$${Pr(I}_{yn}=1)=F\left( \alpha+\rho B_{high}+\lambda'z_{i}+\epsilon_{i} \right)-F\left( \alpha+B_{inital}+\lambda'z_{i}+\epsilon_{i} \right)$$

$${Pr(I}_{yy}=1)=1-F\left( \alpha+\rho B_{high}+{\lambda'z}_{i}+\epsilon_{i} \right)$$

The standard logistic distribution *F( )* with mean zero and variance $\sigma^{2}=(\pi/\sqrt{3)}$^2^ is assumed as it is the most commonly used parametric distribution due to its mathematical tractability [11]. The probability statements reflect the difference in indirect utility achieved from purchasing a vaccine at bid B_i_ and declining the bid. Assigning these probability functions to the data, we calculated the parameter values that maximize the likelihood of observing the WTP bid responses. Given the sample of *n* households, the log-likelihood function takes the form:

$$L=\sum_{i=1}^{n} \left[ I^{nn}\ln\left( F\left( \alpha+{\rho B}_{low}+{\lambda'z}_{i}+\epsilon_{i} \right) \right)+I^{ny}\ln\left( F\left( \alpha+\rho B_{i}+{\lambda'z}_{i}+\epsilon_{i} \right)-F\left( \alpha+B_{low}+{\lambda'z}_{i}+\epsilon_{i} \right) \right)+I^{yn}\ln\left( F\left( \alpha+\rho B_{high}+{\lambda'z}_{i}+\epsilon_{i} \right)-F\left( \alpha+B_{inital}+\lambda'z_{i}+\epsilon_{i} \right) \right)+I^{yy}\ln\left( 1-F\left( \alpha+\rho B_{high}+\lambda'z_{i}+\epsilon_{i} \right) \right) \right]$$

with the ln representing the natural logarithm and $\alpha$ as the intercept. The variable coefficient $\lambda$ represents the marginal effect on WTP taken at the mean of each parameter of interest.

$$\frac{\partial Pr(y=1)}{\partial\lambda}=f\left( \alpha+{\lambda'z}_{i}+\epsilon_{i} \right)\lambda$$

We then estimated the mean WTP for the overall sample (Hanemann, 1984) as:

$$WTP= \frac{1}{\hat{\rho}}(\hat{\alpha}+ \hat{z}^{'} \bar{x})$$

and used the delta method to calculate the confidence intervals around the estimated means [10] (Table S3). The analysis was done in STATA 13 using the doubleb package [13] and then checked in GAUSS 17. Results of the empirical estimation appear in Table S4.

| **Table S1** Descriptions of variables used in analysis | |
| --- | --- |
| Variable | Description |
| Education | Formal education attained by head of household (1=none; 0=some) |
| Income |  |
| *Off-Farm (≤25,000 Tsh)* | Income from off-farm activities in past month (1=yes; 0=no) |
| *Off-Farm (25-100,000 Tsh)* |  |
| *Off-Farm (>100,000 Tsh)* |  |
| *Crops (≤100,000 Tsh)* | Income from crop production in the last season (1=yes; 0=no) |
| *Crops (100-500,000 Tsh)* |  |
| *Crops (>500,000 Tsh)* |  |
| Herd Size | Reported cattle owned by household  Expected milk loss per cow from FMD infection (liters)  FMD in the past year (1=yes; 0=no) |
| Expected milk loss |  |
| FMD in the past year |  |
| Vaccinate | Vaccinated cattle for any purpose in past year (1=yes; 0=no) |
| Use of government vet | Government vet as primary information source (1=yes; 0=no) |
| Cattle sold | Number of cattle sold in past year |
| Vaccine Efficacy | Vaccine presented with 50% efficacy versus 100% (1=yes; 0=no) |
| Outbreak | FMD outbreak at neighbor (1=yes; 0=no) |
| District | Serengeti or Ngorongoro district (1=Serengeti; 0=Ngorongoro) |
| n=432 |  |
|  |  |
|  |  |
|  |  |

| **Table S2** Descriptive statistics for variables used in analysis | | | | |  |
| --- | --- | --- | --- | --- | --- |
| Variable | Mean‡ or  Proportion | Std. Dev. | Median | Min | Max |
| No Formal Education | 0.16 | 0.36 | 0 | 0 | 1.0 |
| Income |  |  |  |  |  |
| *Monthly Off-Farm (≤25,000 Tsh)* | 0.74 | 0.46 | 1.0 | 0 | 1.0 |
| *Monthly Off-Farm (25-100,000)* | 0.13 | 0.34 | 0 | 0 | 1.0 |
| *Monthly Off-Farm (>100,000)* | 0.13 | 0.33 | 0 | 0 | 1.0 |
| *Seasonal Crops (≤100,000 Tsh)* | 0.67 | 0.47 | 1.0 | 0 | 1.0 |
| *Seasonal Crops (100-500,000)* | 0.16 | 0.36 | 0 | 0 | 1.0 |
| *Seasonal Crops (>500,000)* | 0.18 | 0.38 | 0 | 0 | 1.0 |
| Herd Size‡ | 42 | 59 | 20.0 | 1.0 | 530 |
| Expected Milk Loss (in liters per cow) ‡ | 0.70 | 0.59 | 0.61 | 0 | 5.0 |
| Cattle sold in the past year‡ | 5.9 | 6.7 | 2.0 | 0 | 21 |
| FMD experienced in past year | 0.69 | 0.46 | 1.0 | 0 | 1.0 |
| Vaccinated for any cattle disease in past year | 0.19 | 0.40 | 0 | 0 | 1.0 |
| Use government vet | 0.34 | 0.48 | 0 | 0 | 1.0 |
| Vaccine efficacy of 50% | 0.45 | 0.49 | 0 | 0 | 1.0 |
| Male head of household | 0.84 | 0.36 | 1.0 | 0 | 1.0 |
| 50% efficacy*male head of household | 0.60 | 0.49 | 0 | 0 | 1.0 |
| Serengeti district | 0.59 | 0.49 | 1.0 | 0 | 1.0 |
| Outbreak @ Neighbor | 0.57 | 0.50 | 1.0 | 0 | 1.0 |
| n=432 |  |  |  |  |  |

| **Table S3** Estimated WTP averages with 95% CI | | | |
| --- | --- | --- | --- |
|  | Mean | Lower Bound | Upper Bound |
| Routine | 3887 | 2555 | 5217 |
| Emergency | 5411 | 3551 | 7271 |
| USD 1.00=2100 Tanzanian shillings | | | |

| **Table S4** Vaccination Determinants | | | | |
| --- | --- | --- | --- | --- |
| Variable | Routine Marginal Effects  (CI 95%) | P value | Emergency Marginal Effects  (CI 95%) | P value |
|  |  |  |  |  |
| Education (0=Formal; 1=No Formal) | 681 (-7,1356) | 0.096 | 655 (-369,1679) | 0.295 |
| Income |  |  |  |  |
| *Off-Farm (≤25,000 Tsh)* | Base Case | | | |
| *Off-Farm (25-100,000)* | 589 (-34,1213) | 0.119 | 1962 (835,3090) | 0.004 |
| *Off-Farm (>100,000)* | 1022 (360,1685) | 0.010 | 1763 (672,2854) | 0.007 |
| *Crops (≤100,000 Tsh)* | Base Case | | | |
| *Crops (100-500,000)* | 1635 (806,2465) | 0.001 | 2294 (1034,3554) | 0.003 |
| *Crops (>500,000)* | -445 (-1067,176) | 0.237 | -403 (-1513,3554) | 0.552 |
| Herd Size† | 26 (-192,243) | 0.846 | 42 (-348,432) | 0.859 |
| Expected Milk Loss (in liters per cow) | 306 (-94,707) | 0.207 | 423 (-205,1051) | 0.270 |
| Cattle sold in past year | 36 (-.33,71) | 0.096 | 11 (-48,71) | 0.753 |
| FMD experience in past year (0=No; 1=Yes) | -241 (751,270) | 0.439 | -283 (-1156,590) | 0.595 |
| Vaccinated for any cattle disease in past year (0=No; 1=Yes) | -247 (-795,299) | 0.457 | 216 (-754,1186) | 0.715 |
| Use of government vet (0=No; 1=Yes) | -663 (-1113,-214) | 0.014 | -1817 (-2626,-1008) | 0.001 |
| Vaccine efficacy (0=100%; 1=50%) | 1573 (370,2778) | 0.031 | 2318 (107,4529) | 0.085 |
| Gender (0=Female; 1=Male) | 1031 (321,1740) | 0.016 | 857 (-478,2192) | 0.292 |
| Gender*efficacy (0=100%; 1=50%) | -1458 (-2740,-174) | 0.060 | -2737 (-5066,406) | 0.053 |
| District (0=Ngorongoro; 1=Serengeti) | -270 (-751,212) | 0.358 | 94 (-779,967) | 0.860 |
| Outbreak (0=Village; 1=@Neighbor) |  |  | -476 (-1244,293) | 0.314 |
| Log Likelihood | -415 |  | -498 |  |
| Chi-2 Statistic | 39.09 |  | 41.87 |  |
| † Log of variable  USD 1.00=2100 Tanzanian shillings |  |  |  |  |
|  | | | | |

**References**

[1] Sallu R, Kasanga CJ, Mathias M, Yongolo M, Mpelumbe-Ngeleja C, Mulumba M, et al. Molecular survey for foot-and-mouth disease virus in livestock in Tanzania, 2008–2013. Onderstepoort J Vet Res 2014;81:2008–13. doi:10.4102/ojvr.v81i2.736.

[2] Grubman MJ, Baxt B. Foot-and-mouth disease. Clin Microbiol Rev 2004;17:465–93. doi:10.1128/CMR.17.2.465.

[3] Parida S. Vaccination against foot and mouth disease virus: strategies and effectiveness. Expert Rev Vaccines 2009;8:347–65.

[4] Alolayan MA, Evans JS, Hammitt JK. Valuing Mortality Risk in Kuwait: Stated-Preference with a New Consistency Test. Environ Resour Econ 2017;66:629–46. doi:10.1007/s10640-015-9958-1.

[5] Goldberg I, Roosen J. Scope insensitivity in health risk reduction studies: A comparison of choice experiments and the contingent valuation method for valuing safer food. J Risk Uncertain 2007;34:123–44. doi:10.1007/s11166-007-9006-9.

[6] Hanemann M, Loomis J, Kanninen B. Statistical Efficiency of Double-Bounded Dichotomous Choice Contingent Valuation. Am J Agric Econ 1991;73:1255–63. doi:10.2307/1242453.

[7] Alberini A. Efficiency vs bias of willingness to pay estimates: bivariate and interval data models. J Environ Econ Manage 1995;29:169–80.

[8] Boyle KJ, Bishop RC, Welsh MP. Starting Point Bias in Contingent Valuation Bidding Games. Land Econ 1985;61:188–94. doi:10.2307/3145811.

[9] Knight-Jones TJD, Rushton J. The economic impacts of foot and mouth disease - What are they, how big are they and where do they occur? Prev Vet Med 2013;112:162–73. doi:10.1016/j.prevetmed.2013.07.013.

[10] Greene WH. ECONOMETRIC ANALYSIS. 5th ed. New Jersey: Prentice Hall; 2003.

[11] Kerr GN. Dichotomous choice contingent valuation probability distributions. Aust J Agric Resour Econ 2000;44:233–52. doi:10.1111/1467-8489.00109.

[12] Hanemann MW. Welfare Evaluations in Contingent Valuation Experiments with Discrete Responses. Am J Agric Econ 1984;66:332–41. doi:10.2307/1240800.

[13] López-Feldman A. Introduction to Contingent Valuation Using Stata Contingent Valuation ( I ) 2013.
