# Supplementary material for: Spatial and temporal risk as drivers for adoption of foot and mouth disease vaccination
Source: Vaccine. 2018 Aug 9;36(33):5077–83. doi: 10.1016/j.vaccine.2018.06.069 (PMC6073883; doi:10.1016/j.vaccine.2018.06.069)
Supplement: Supplementary Data 2 [file mmc2.docx]

Data Set Name

FMDvaccination_data.xlsx

Caption

432 observations across 26 variables

Description

For emergency vaccination, ‘ebid1’ is 1=yes if household responded yes to the bid in ‘Ebidprice’ and 0 otherwise. ‘E2’ is 1=yes if the household responded yes to the second bid in ‘EBid2’. Enter ‘id’ followed by these four columns, order as is, for the dependent variable. The last five columns correspond to the routine vaccination scenario in this format. Others enter as independent variables. Use data as is with Gauss code or follow Doubleb package instructions in Stata.

Data and code available upon request.
